# There Is No Relation between Epitympanic Recess Volume and Chronic Otitis Media

**DOI:** 10.3390/tomography9040106

**Published:** 2023-07-08

**Authors:** Fatma Dilek Gokharman, Omer Kocak, Baris Irgul, Pinar Kosar, Sonay Aydin

**Affiliations:** 1 Department of Radiology, Ankara Training and Research Hospital, Ankara 06660, Turkey; omerkocak994@gmail.com (O.K.); pikosar@gmail.com (P.K.); 2 Department of Radiology, Erzincan Binali Yidirim University, Erzincan 24100, Turkey; barisirgul@gmail.com (B.I.); sonay.aydin@erzincan.edu.tr (S.A.)

**Keywords:** otitis media, ear infection, Eustachian tube, middle ear

## Abstract

Background: Chronic otitis media is recurrent infection of the middle ear and mastoid air cells in the setting of perforation of the tympanic membrane. Risk factors for chronic otitis media include predisposing characteristics such as gender, allergies, Eustachian tube dysfunction, history of acute otitis media, and upper respiratory tract infection. The purpose of this study was to evaluate the potential relationship between chronic otitis media and epitympanic recess volume. Materials and Methods: A total of 197 patients with chronic otitis media had their epitympanic recess volume compared to the epitympanic volume of 99 healthy controls. The epitympanic recess volume was measured via the 3D volumetric measurement tool of the local PACS. Epitympanic recess volume measurement was performed using axial sections in a plane starting from the level of the malleus head–anvil body in the craniocaudal direction to the tegmen tympanum. Results: It was shown that patients with bilateral involvement had an epitympanic recess volume of 75.00 mm^3^, compared to 72.30 mm^3^ in those with unilateral chronic otitis media. The healthy control group’s median value for the epitympanic recess was 74.73 mm^3^. Conclusions: Epitympanic volume values did not differ substantially between patients with chronic otitis media and healthy persons, and epitympanic volume was not recognized as a predisposing factor (*p* = 0.686).

## 1. Introduction

Chronic otitis media is a common condition that affects the middle ear and mastoid air cells. It is an inflammatory disease which influences the various middle ear tissues and can be recurrent if not properly treated. Risk factors for chronic otitis media include a history of acute otitis media, upper respiratory tract infections, gender (with females having an increased risk), allergies, and Eustachian tube dysfunction [1].

Tomographic imaging can help to diagnose chronic otitis media as it may reveal nonspecific soft tissue values at the middle ear level or calcification/retraction appearance in the tympanic membrane. Additionally, the erosion of ossicles or wall components may also be observed during imaging scans which could indicate advanced cases of this condition [2]. Treatment typically involves antibiotics, but surgery might also be necessary if there are complications such as hearing loss due to fluid buildup in the inner ear [3].

Infections that begin in the middle ear can extend to the mastoid section of the temporal bone, causing mastoiditis, which can lead to the development of a subperiosteal abscess. Pus in the mastoid cavity may occasionally escape from the mastoid tip infero-medially and travel down to produce a Bezold’s abscess in the neck muscles.

The facial nerve runs through the temporal bone, and it is conceivable for the middle ear to spontaneously dehisce. Cholesteatoma can also destroy the bone covering of the facial nerve, leading to facial palsy as a consequence of local inflammation and infection. Cholesteatoma may also impact the skeletal labyrinth. The lateral semicircular canal is the most frequent site of erosion, which can result in imbalance or vertigo. The fifth and sixth cranial nerves have the potential to be affected by an infection at the tip of the temporal bone. This disorder is known as gradenigo syndrome, and it includes retroorbital pain on the same side, abducens nerve dysfunction, and otorrhea [4,5].

Extracranial complications have been mentioned so far. Meningitis, extradural abscess, subdural abscess, brain abscess, sigmoid sinus thrombosis, and otitic hydrocephalus can be given as examples of intracranial complications. Intracranial problems may emerge as a result of COM owing to infection transfer via a variety of mechanisms. Cholesteatoma can directly erode the skull base, resulting in extradural or subdural abscess development. Via local venous pathways, an infection can spread to the sigmoid sinus, resulting in sigmoid sinus thrombosis. From the sinus, septic emboli can migrate, causing infection and sepsis to disseminate widely. Inflammation of the ventricles is a rare but severe intracranial complication of COM. Otitic hydrocephalus occurs when cerebrospinal fluid outflow is obstructed, resulting in elevated intracranial pressure. Meningitis is caused by hematogenous spread to the meninges, and infection can extend into the brain via emissary veins, creating intracerebral (temporal lobe) or cerebellar abscesses.

When treating individuals with COM who appear with headaches, fevers, or any new neurological indications, a strong index of suspicion is required. CT or MRI are used to confirm the diagnosis, and broad-spectrum IV antibiotics should be administered immediately. A comprehensive neurological examination and neurosurgical opinion should be conducted. Mastoidectomy can be utilized to evacuate extradural abscesses, whereas neurosurgeons are required to treat subdural and brain abscesses.

People who experience chronic otitis symptoms, such as pain or drainage from the ear, should seek medical attention immediately so that they do not develop more severe problems in the future. Early diagnosis and treatment will improve the prognosis for those affected by this disease. Although the otologist is the best person to treat chronic otitis media, the first treating physician plays an important role in diagnosis, evaluation, and therapy [6,7].

A number of anatomical characteristics, such as middle ear cavity volume, aditus ad antrum diameter, and Eustachian tube diameter, have been identified as risk factors for the development of COM, according to recent studies [6,7,8]. The primary objective of the current study is to determine the potential role of epitympanic recess volume, an additional anatomical variable, in the development of chronic otitis media.

The epitympanum, also known as the epitympanic recess, is a cavity in the dorsal (tegmental) wall that houses the malleus and incus ossicles. It is found in the topmost region of the tympanic cavity. The epitympanum connects to the mastoid antrum posteriorly via the aditus ad antrum and subsequently through the mastoid air cells. Its principal components are the malleus head, incus body, and short prominence, which articulate with each other at the incudomalleolar joint. On axial computed tomography (CT), the normal appearance of these structures results in an ice cream cone sign [9].

Anterior to the epitympanic recess is a separate cavity, the anterior epitympanic recess, from which the rest of the epitympanum is separated. This space is also known as the supratubal recess.

Ventilation of the middle ear is also essential for successful surgical reconstruction in cases of chronic otitis media [10]. Anterior epitympanic recess (AER) has attracted attention as a possible additional ventilation route in recent years. The anterior epitympanic recess configuration is easily affected by persistent stapedial artery, facial nerve schwannomas, hemangiomas in the geniculate region of the facial nerve canal, and congenital and acquired cholesteatomas [9].

In previous studies, the role of anterior epitympanic recess volume in the formation of chronic otitis media has been investigated. A significant difference was found between normal ears and ears with cholesteatoma in terms of anterior epitympanic recess size, but no relationship was found between anterior epitympanic recess size and pneumatization of mastoid cells in ears with cholesteatoma. No significant difference was found between normal ears and ears with chronic otitis media in terms of anterior epitympanic recess size [11].

When we searched the literature, it was seen that the relationship between anterior epitympanic recess volume and chronic otitis media was investigated [1,6,7], but the relationship between epitympanic recess volume and chronic otitis media has not been investigated before. As a result, the purpose of this study was to look into the association between epitympanic recess volume and chronic otitis media.

## 2. Materials and Methods

This study included patients who have been followed up with a chronic otitis media diagnosis between 1 June 2021 and 31 August 2021. Also, healthy controls with no clinical diagnosis were included.

Exclusion criteria from the study were as follows: maxillofacial anomaly; temporal bone fracture; history of mastoidectomy; being younger than 18 years of age; or have a CT image with artifacts that do not allow the specified measurements to be made.

A Philips Brilliance 16-detector MDCT system (Philips Medical Systems, Cleveland, OH) was used to create CT images. Imaging parameters were as follows: 120 kV, 105 mA, 0.8 mm slice thickness, 0.4 mm interval, 0.75 s rotation time, 16 × 0.75 collimation, and a 512 × 512 matrix. Axial images of 1 mm slice thickness were used to generate coronal and sagittal images. All three planes were utilized to perform the measurements and collect the data.

**Figure 1:**
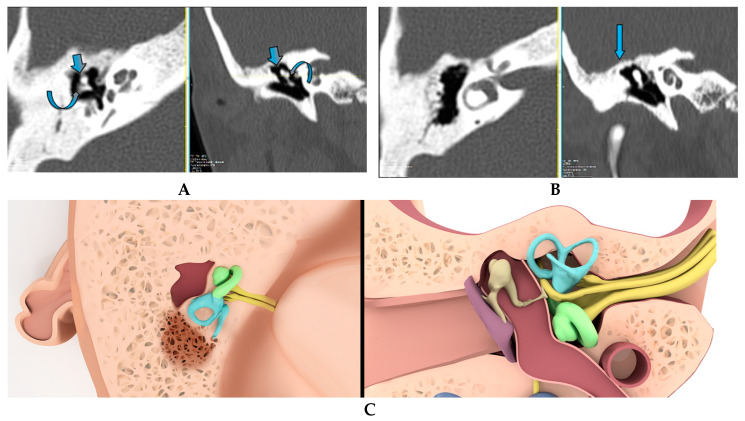

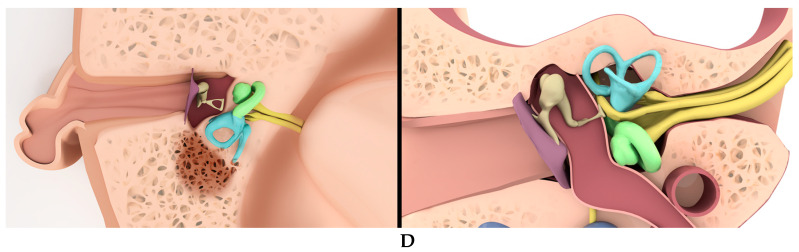
Axial (**A**) and coronal (**B**) section view and corresponding illustrations (**C**-axial, **D**-coronal) of temporal bone at the level of malleus head (**A**, **C** Straight thick arrow)-incus body section (**A**, **C** Curved arrow) and at the level of tegmen tympani (**B**, **D** Straight thin arrow) (Axial sections are cross-sectional images at the level of the straight line in the coronal section).

**Figure 2:**
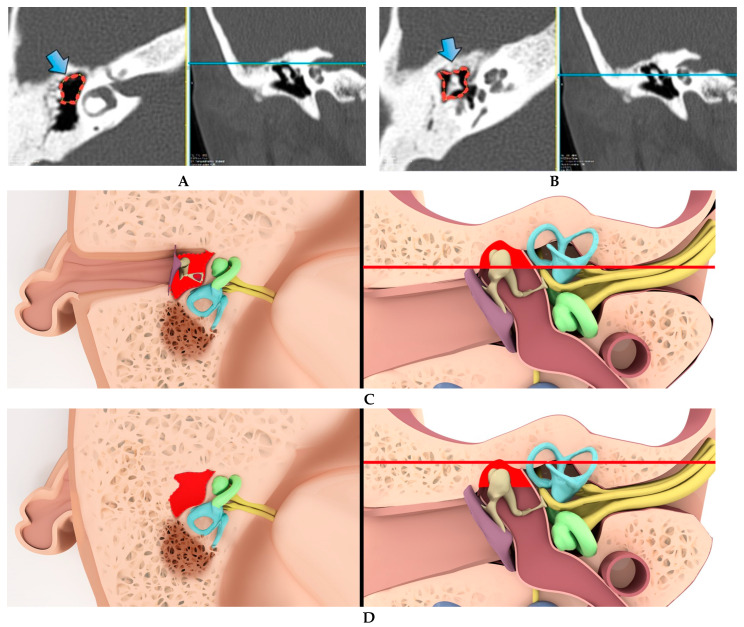
Volumetric marking of the boundaries of the caudal and cranial levels of the epitympanic recess (thick arrows) on axial (**A**) and coronal (**B**) CT images and corresponding illustrations (**C**-axial, **D**-coronal) (Axial slices are cross-sectional images at the level of the straight line in the coronal section).

The epitympanic recess volume was measured via 3D volumetric measurement tool of the local PACS. Epitympanic recess volume measurement was performed using axial sections in a plane starting from the level of the malleus head and body in the craniocaudal direction to the tegmen tympanum. The mentioned levels were defined by using coronal sections (Figure 1 and Figure 2).

Moreover, characteristics such as unilateral/bilateral involvement, presence or absence of addus ad antrum involvement, presence or absence of mastoid cellular involvement, degree of mastoid cellular involvement, and patient history of surgery were investigated.

The degree of involvement of the mastoid cells was evaluated as less than 1/3 of this level as mild, less than 2/3 as moderate, and more than 2/3 as severe.

Our study was approved by the ethics committee. The ethics committee approval number is as follows: EBYU—KAEKE—1845362.11.ED.28451.

All procedures performed in studies involving human participants were in accordance with the ethical standards of the institutional and/or national research committee and with the 1964 Helsinki declaration and its later amendments or comparable ethical standards.

### Statistical Analysis

The Shapiro–Wilk test was used to determine the normality of numerical quantities. Student’s t-test will be used to compare regularly distributed values between the two groups, and the Mann–Whitney *U* test will be used to examine non-normally distributed data.

Categorical analyses were carried out using the Chi-square test and, if necessary, the Fisher exact test. For numerical variables, descriptive statistics were evaluated as meansd, median [min max], and numbers and percentages (%) for categorical variables.

For statistical analysis, the SPSS Windows 23.0 (IBM corp., NY, USA) package program was utilized, and *p* values were considered statistically significant.

## 3. Results

We conducted our study with a total of 321 individuals, but we excluded 25 individuals from the study. After the 25 individuals were excluded from the study, 296 individuals were included in the study. There were various reasons why 25 individuals were excluded from the study.

Four individuals were excluded because they had maxillofacial anomalies. Temporal bone fracture was detected in two individuals, and they were excluded from the study, because temporal bone fractures could affect the accuracy of the measurements. Eight individuals were excluded because they had a history of mastoidectomy. In addition, images were excluded from the study because of low diagnostic quality due to movement anomalies in 11 individuals.

A total of 197 patients with chronic otitis media and 99 healthy individuals were included in the study.

Of the individuals participating in our study, 160 were women and 136 were men. The age distribution of women was found to be a minimum of 18, a maximum of 72, and a mean age of 52.6 ± 8.32. The age distribution of men was found to be a minimum of 20, a maximum of 69, and a mean age of 51.2 ± 6.41. Of the 25 individuals excluded from the study, 14 were female and 11 were male.

Unilateral involvement was observed in 165 of patients with chronic otitis media and bilateral involvement was observed in 32 patients. In addition, involvement of the aditus ad antrum was observed in 123 patients, and this level of involvement was not observed in 74 patients.

Mastoid cellular involvement was not observed in 25 patients, it was evaluated as mild involvement in 134 patients, moderate involvement in 27 patients, and severe involvement in 131 patients. A total of 9 of 197 patients with chronic otitis media had a history of surgery and findings, and 188 had no history of surgery (Table 1).

The epitympanic recess volume descriptive statistics of 197 cases diagnosed with chronic otitis media are summarized in Table 1. The mean epitympanic recess volume of unilateral COM cases was 77.06 ± 24.67 mm^3^, and the median was 72.30 mm^3^, varying between 41.45 and 145.96 mm^3^. In patients with bilateral involvement, the mean volume was calculated as 76.61 ± 17.66 mm^3^, and the median was 75.00 mm^3^, varying between 52.86 mm^3^ and 114.92 mm^3^ (Table 2).

Epitympanic recess volumes of unilateral and bilateral chronic otitis media cases were compared. There was no statistically significant difference between the values of unilateral epitympanic recess volume at 72.30 mm^3^ [41.45–145.96] and bilateral epitympanic recess volume at 75 mm^3^ [52.85–114.92] (*p* = 0.682) (Table 2).

There was no statistically significant difference in the distribution of other clinical features of unilateral cases and bilateral cases of chronic otitis media (*p* > 0.05) (Table 2).

The mean epitympanic recess volume of the healthy control group (*n* = 99) was calculated as 83.125 ± 43.625 mm^3^ and the median value was 74.73 mm^3^.

No statistically significant difference existed between the epitympanic recess volumes of unilateral or bilateral COM cases and those of healthy controls (*p* = 0.686) (Table 3).

## 4. Discussion

The anterior epitympanic recess, also called the supratubal recess, is a narrow space in the epitympanum anterior to the malleus. The pinion separates it from the epitympanum proper. The anterior epitympanic recess contains the three structural categories listed below: A, the main cavity seen above the tensor tympanic fold; B, the recess divided into two cavities above and below the TTF; and C, the recess adjacent to the Eustachian tube. In 48 human temporal bones, several distances related to each form of recess were measured. The following distances were increased significantly between types A, B, and C: length of the TTF; distance between the top of the cavity beneath the TTF and the inferior border of the tensor tympanic semicanal; and distance from the pinion to the anterior wall of the recess.

In addition, the intact canal wall method was used to create a pathway from the epitympanum to the Eustachian tube in 12 human temporal bones. By removing the pinion and TTF from type C, the route could be expanded greatly. In cases of short TTF, removal of the bony plate to which the TTF was affixed was also required for types A and B. During surgical intervention, it is crucial to consider the structural variations of the anterior epitympanic recess and the location of its adjacent tissues. The recess should be conducted according to the method best suited to each recess type [12].

The persistence of the lamina between the squamous and petrous portions of the temporal bone may result from an interruption during the temporal bone’s development. At the level of the antrum, the petrosquamous lamina (Köerner’s septum) is a dense bony lamina that divides the mastoid cavity into superficial squamous and deeper petrosal portions. It has been hypothesized that this structure is the consequence of a failure in temporal bone development. It is well known that Köerner’s septum can complicate mastoid surgery, that a false mastoid antrum may be discovered, and that the facial nerve may be easily damaged as a consequence. Multiple investigations on the relationships between the Koerner’s septum and numerous temporal bone structures have been published in the past. Köerner’s septum may also be associated with the cog process, the middle bone portion of the petrosquamous lamina. Considering that the longer the cog process, the higher the risk of retraction pockets and/or cholesteatoma due to epitympanum obstruction, Köerner’s septum may also have clinical implications in relation to retraction pockets and/or cholesteatoma. Temporal bones with Köerner’s septum were found to have a longer cog process and a smaller anterior epitympanic recess [13].

As these anatomical structures serve as landmarks for the attic, the anterior epitympanic plate (cog) and Köerner’s septum have gained significance since the advent of transcanal mastoidectomy. Moreover, various morphological types of cog and Köerner’s septum revealed an embryological relationship to the development of the isthmic membrane; the latter is linked to aeration of the attic and consequently influences the pathological development of the cholesteatoma. The difference in distribution of different morphological types of anterior epitympanic plates, “cog,” as well as the difference in the presence of Köerner’s septum between cholesteatomatous and non-cholesteatomatous chronic suppurative otitis media, are proposed as risk factors for the development of cholesteatoma and may predict a cholesteatoma on CT images [14].

Anterior epitympanic recess has been included in the literature with different studies due to its important anatomical neighborhoods and frequent association with cholesteatoma in previous studies [9]. It has been determined that the anterior epitympanic recess bone structure is rarely affected by inflammatory processes and there is no relationship between the recess volume and the pneumatization of mastoid cells [10,11]. However, a significant difference was found between normal ears and ears with cholesteatoma in terms of anterior epitympanic recess size [6,11].

A wide variety of pathological processes can affect the appearance of the anterior epitympanic recess. Examples of these pathologies are facial nerve schwannoma, facial nerve canal hemangioma, persistent stapedial artery, and cholesteatoma formation. The anterior epitympanic recess can be easily deformed due to pathologies at this level [9,11].

In previous studies, no significant relationship was found between anterior epitympanic recess volume and chronic otitis media [1,11]. There are studies on anterior epitympanic recess in the literature, but it has been observed that studies on epitympanic recess are few. The purpose of this study was to evaluate the potential relationship between chronic otitis media and epitympanic recess volume.

In previous studies, significant differences were observed in the middle ear volume and in the structures connecting the middle ear with the mastoid cellular and oral cavity such as the aditus ad antrum and the Eustachian tube [1,7,8,10,11]. The association between aditus ad antrum diameter measurement and chronic otitis media and accompanying illnesses (tympanosclerosis and myringosclerosis) was explored in this study, because the middle ear cavity is ventilated through the aditus ad antrum. In this study, temporal CT scans of 162 people were reviewed retrospectively, and the internal diameter of the aditus was determined in axial sections. The diameters of sick and healthy ears were compared. In healthy people, women had a smaller diameter. The diameter was shown to be lower in instances of otitis media and tympanosclerosis than in healthy persons, albeit there was no statistically significant outcome in all cases. As a result, the fact that the aditus ad antrum is narrower in infected ears suggests that it may contribute to the development of otitis media [7].

Another study looked into the function of Eustachian tube diameter in chronic otitis media. This research comprised 154 individuals who had unilateral chronic otitis media. Axial slices were used to generate CT images. In the ill ear, statistical analysis revealed a markedly small Eustachian tube diameter [8].

Another study investigated the relationship between chronic otitis media and middle ear volumes. In this study, 144 individuals were included and middle ear volumes of healthy individuals and individuals with chronic otitis media were compared. As a result of the study, a decrease in the middle ear volume on the diseased side was found in individuals with chronic otitis media. And it has been shown to cause a decrease in middle ear volumes in the ear affected by chronic otitis media [6].

In the current study, we only examined the middle ear volume in terms of the level of epitympanic recess.

In individuals with unilateral chronic otitis media, the median epitympanic recess volume was 72.30 mm^3^, and the median value in those with bilateral involvement was 75.00 mm^3^. In the healthy control group, the median value of the epitympanic recess volume was 74.73 mm^3^. There was no significant difference in epitympanic volume levels between chronic otitis media patients and healthy persons. We did not detect epitympanic volume as a predisposing feature.

Among the limitations of the study is its retrospective character. Increasing the number of populations may also lead to different results. Epitympanic recess measurements were made by a single investigator at one time. Therefore, inter- and intraobserver variability data cannot be presented. Differentiating the cases as otitis media chronica mesotympanalis and otitis media chronica epitympanalis may alter the results, but we do not have enough cases to execute such a classification, and the statistical significance would be lost after the grouping. In addition, we wanted to refer to studies that previously investigated the relationship between ethnic origins and chronic otitis media. We also considered measuring the variation of epitympanic recess volume across ethnicities. However, the ethnic distributions of the 296 individuals included in the study consisted of similar groups. For this reason, we could not provide any data on ethnic origins, because significant statistical data could not be obtained.

## 5. Conclusions

In conclusion, our study is the first to measure epitympanic recess volume in normal individuals and cases with chronic otitis media. According to our first and preliminary results, it has been shown that the presence of chronic otitis media does not cause a significant change in the anatomy and volume of the epitympanic recess. Further prospective studies with larger populations might alter the results.

## Figures and Tables

**Table 1:** Comparison of other clinical features of bilateral and unilateral chronic otitis media cases.

	Unilateral	Bilateral	
	*n* (%)	*n* (%)	
Aditus ad antrum involvement	98 (59.4)	25 (78.1)	0.045
No aditus ad antrum involvement	67 (40.6)	7 (21.9)	
Mild mastoid cellular involvement	14 (8.5)	0 (0)	0.112
Moderate mastoid cellular involvement	20 (12.1)	7 (21.9)	
Severe mastoid cellular involvement	108 (65.5)	23 (71.9)	
No mastoid cellular involvement	23 (13.9)	2 (6.3)	
Has a history of surgery	9 (5.5)	0 (0)	0.176
No history of surgery	156 (94.5)	32 (100)	

*p* value was obtained from Chi-square test.

**Table 2:** Comparison of epitympanic recess volumes in bilateral and unilateral chronic otitis media cases and healthy control group.

	Unilateral M [Min–Max]	Bilateral M [Min–Max]	Healthy Control Group	*p*
Epitympanic recess volume	72.30 [41.45–145.96]	75.00 [52.85–114.92]	74.73 [39.49–126.76]	0.682

*p* value was obtained from Mann–Whitney *U* test. M: Median.

**Table 3:** Descriptive statistics of epitympanic recess volume of chronic otitis media cases and healthy control group.

	*n*	Mean ± Sd	M [Min–Max]
Epitympanic recess volume (unilateral)	165	77.06 ± 24.67	72.30 [41.45–145.96]
Epitympanic recess volume (bilateral)	32	76.61 ± 17.66	75.00 [52.85–114.92]
Epitympanic recess volume (healthy control group)	99	84.125 ± 43.625	74.73 [39.49–126.76]

M: Median.

## Data Availability

The data that support the findings of this study are available from the corresponding author (F.D.G.), upon reasonable request.
